# Toll-like receptor-mediated innate immune response correlate with the pathogenicity of *Eimeria tenella* infection in SPF chickens

**DOI:** 10.1186/s13071-026-07466-2

**Published:** 2026-05-27

**Authors:** Huili Zhu, Guijie Zheng, Danni Wang, Qian Zhang, Yanhui Han, Song Wang, Qiangqiang Wang, Hongxuan He, Longxian Zhang, Jianhe Hu

**Affiliations:** 1 College of Animal Science and Veterinary Medicine, Henan Institute of Science and Technology, Xinxiang, 453003 Henan People’s Republic of China; 2 Ministry of Education Key Laboratory for Animal Pathogens and Biosafety, Zhengzhou, 453000 Henan People’s Republic of China; 3 Yebio Bioengineering Co., Ltd., of Qingdao, Qingdao, 266108 Shandong People’s Republic of China; 4 College of Animal Science and Veterinary Medicine, Henan Agricultural University, Zhengzhou, 450046 Henan People’s Republic of China

**Keywords:** *Eimeria tenella*, Innate immunity, Pathogenicity, Correlation, Chicken

## Abstract

**Background:**

*Eimeria tenella*, one of the most virulent causative agents of coccidiosis, specifically colonizes the cecum and imposes substantial economic losses on the poultry industry. Toll-like receptors (TLRs), key mediators of the innate immune response, initiate rapid defense against invading parasites. However, their roles during *E. tenella* infection remain poorly defined.

**Methods:**

In this study, coccidiosis infection parameters (cecal lesions, oocyst output, and clinical signs) were monitored, and the expression profiles of 10 chicken TLRs (chTLRs) and related immune genes in the cecum and spleen of *E. tenella*-infected chickens were characterized at multiple time points using quantitative real-time polymerase chain reaction (PCR) and histopathological analysis. Spearman correlation analysis was performed to evaluate associations between TLR expression and pathogenicity parameters.

**Results:**

Infected chickens exhibited marked clinical signs, although no mortality occurred in either group. Cecal lesions became apparent by 96 h post-infection (hpi), peaking at 144 hpi, and were accompanied by significant epithelial necrosis and extensive inflammatory cell infiltration. Most TLR genes showed distinct expression patterns between the cecum and spleen: cecal TLRs were upregulated during the middle phase (24–72 hpi) of infection, whereas splenic TLRs were downregulated in later stages (96–168 hpi). Key proinflammatory cytokines—interleukin (IL)-1β, tumor necrosis factor (TNF)-α, and interferon (IFN)-γ—were elevated in the cecum during early infection but declined in the spleen during later phases. Infection also induced dynamic expression changes in the downstream signaling molecules myeloid differentiation factor 88 (MyD88) and nuclear factor-kappa B (NF-κB) p65 in both tissues. Spearman correlation analysis revealed that, in the cecum, chTLR7 positively correlated with both oocyst output and lesion scores, whereas chTLR2a positively correlated with lesion scores. By contrast, chTLR1a, chTLR1b, chTLR15, and chTLR21 negatively correlated with oocyst output. In the spleen, chTLR15 and chTLR21 positively correlated with oocyst output, while chTLR5 uniquely and negatively correlated with lesion scores.

**Conclusions:**

These findings identify both proinflammatory and potentially protective TLR pathways in subclinical coccidiosis.

**Graphical abstract:**

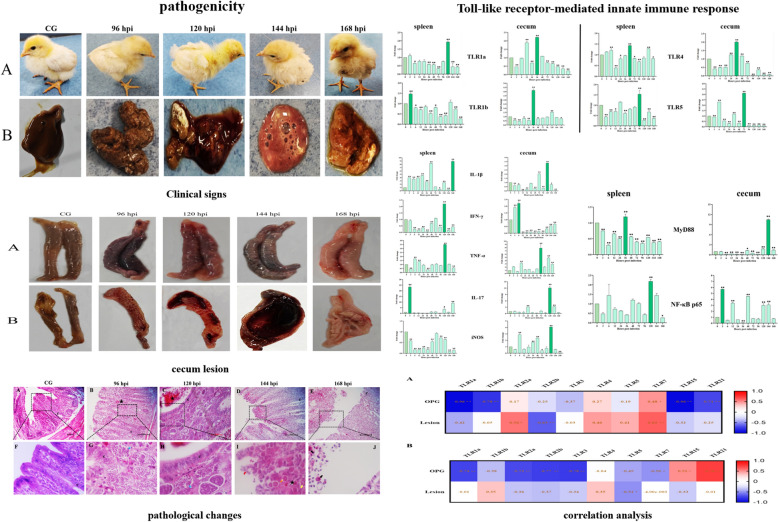

**Supplementary Information:**

The online version contains supplementary material available at 10.1186/s13071-026-07466-2.

## Background

Chicken coccidiosis is a serious parasitic disease caused by seven species of *Eimeria* that infect the intestine tract of chickens, leading to substantial economic loss in the poultry industry [1]. The disease is associated with increased feed conversion ratios, reduced weight gain, clinical infections, and mortality in persistently infected flocks, with global annual production losses and control costs estimated at US$ 14 billion [2]. Among these species, *Eimeria tenella* is one of the most pathogenic, and it specifically colonizes the cecum,which can cause symptoms ranging from bloody diarrhea and weight loss to death [3]. For decades, control strategies relying on anticoccidial drugs have been largely successful. However, these approaches are increasingly compromised by issues such as drug resistance, food safety concerns, and high production costs [4]. Current understanding of the avian immune response to *Eimeria* has primarily emphasized adaptive immunity, with limited research dedicated to innate immune mechanisms [5].

Innate immunity serves as the first line of host defense against pathogen invasion. Previous studies have demonstrated a positive correlation between pathogenic infection and the expression levels of immune-related genes—including Toll‑like receptors (TLRs), cytokines, and antimicrobial peptides—in specific infected tissues, which suggesting a functional role for these proteins in the host immune response to infection [6]. For instance, chickens infected with Fowl adenovirus serotype 8b (FAdV‑8b) exhibited significant upregulation of chicken Toll‑like receptor (chTLR)2 and chTLR15 in the spleen, along with elevated interleukin (IL)‑2 and reduced interferon (IFN)‑γ levels during the early phase of infection, when viral load was detectable in most tissues. Viral shedding occurred as early as 3 days post‑infection (dpi) and peaked at 7 dpi [7]. In response to avian influenza viruses, H9N2-PB2 reassortant H5N1 virus and nonreassortant H5N1 virus induced dysregulation of cytokines, such as IL-1, IFN, IL-2, and IL-6, in lungs and brain might have contributed to the pathogenesis of both the viruses in mice [8]. In addition, the dog infected with Visceral leishmaniosis showed that increased parasite load leads to upregulation of TLR-2, IL-10 and TNF-α, indicating that abundance of these transcripts is associated with infection [9]. Previous studies also demonstrated that *E. tenella-*challenged chickens exhibited marked upregulation of chTLR3, 5, 7, 15 along with early IFN-γ expression in the bursa, whereas nearly all ten chTLRs were significantly elevated in the cecal tonsil during later infection stages, with corresponding changes in IFN-γ expression, indicating the potential role of these molecules in host immune responses against *E. tenella* infection [10, 11], However, those studies focused on single tissues and limited time points. How the activation of innate immunity mechanistically influences clinical outcomes during infection remains unclear and is a critical area for investigation.

Therefore, the aim of this study was to investigate the host–pathogen inteaction by simultaneously monitoring disease parameters (cecal lesions, oocyst output, clinical signs) and innate immune gene expression in both cecum and spleen of specific pathogen free (SPF) chickens during *E. tenella* infection. Spearman correlation analysis was used to assess associations between chTLR expression and pathogenicity parameters. The results of this study provide information about the potential influence of host immunity on coccidian infection in chickens.

## Methods

### Animals and parasites

1-Day-old SPF chickens were obtained from a commercial supplier (Xinxiang, Henan, China) and raised under coccidia-free conditions, as previously described [12]. Briefly, all cages, drinkers, and feeders were sanitized prior to housing. Throughout the study until 14 days of age, birds had ad libitum access to coccidiostat-free feed and water to maintain health and welfare. Prior to infection, fecal samples from all birds were microscopically examined to verify the absence of natural coccidial infection. Once confirmed as infection-free, chickens were transferred to another facility and challenged with virulent oocysts. All animal procedures were approved by the Animal Experiments Welfare and Ethics Committee of the Henan Institute of Science and Technology (approval ID: LLSC2024073).

Sporulated oocysts of *E. tenella* were maintained and propagated in susceptible chickens at the Animal Parasitology Laboratory, with passages performed every 3 months. The harvested sporulated oocysts were stored in 2.5% potassium dichromate at 4 °C until use.

### Experimental design

At 14 days of age, 130 chickens were randomly assigned to the infected group and control group (*n* = 65 per group). The infected group received an oral inoculum of 5 × 10^4^ sporulated oocysts in 1 mL phosphate-buffered saline (PBS), whereas the control group received 1 mL of phosphate-buffered saline (PBS) orally [13, 14]. In each group, five birds were assigned to a clinical observation cohort and monitored daily for clinical signs until 168 h post-infection (hpi) without being euthanized. The remaining 60 birds per group formed a sequential sampling cohort. Tissue samples were collected at 12 time points: 1 immediately before infection (0 hpi) and at 11 time points after infection (e.g., 3, 6, 12, 24, 36, 48, 72, 96, 120, 144, and 168 hpi). At each of these time points, five infected and five uninfected control chickens were euthanized for tissue collection. At 0 hpi, the infected and control groups were biologically identical, but both were sampled to maintain a consistent experimental design across all time points. Throughout the study, all chickens were housed under controlled conditions in the animal facility with ad libitum access to commercial feed and water.

### Clinical signs, lesion scoring, and histopathology analysis

Clinical signs and mortality were recorded daily throughout the experimental period. At each designated time point (0, 3, 6, 12, 24, 36, 48, 72, 96, 120, 144, and 168 hpi), five chickens from each group (infected and control) were randomly selected and euthanized for tissue collection. From each bird, both ceca were collected. Cecal lesion scoring was performed immediately on the intact ceca according to the method of Johnson and Reid [15], using a scale from 0 (no lesion) to 4 (severe lesion), based on morphological changes such as cecal wall thickening or shrinkage and the presence of bloody contents. After scoring, the ceca were opened longitudinally. The contents from both ceca were pooled for oocyst counting. One cecum was then used for histopathological examination (fixed in 10% formalin), while the other cecum was snap-frozen in liquid nitrogen and stored at −70 ℃ for subsequent RNA extraction and gene expression analysis.

### Oocyst assessment

For oocyst assessment, cecal contents were collected at each euthanasia time point (0, 3, 6, 12, 24, 36, 48, 72, 96, 120, 144, and 168 hpi). At each time point, both ceca were collected from each bird. Cecal contents from both ceca were pooled, homogenized, and then examined for oocyst counts. Oocyst counts were performed as described by Jordan et al. [16]. Briefly, 5 g of pooled cecal content was mixed with 15 mL of water, homogenized, and loaded onto a hemacytometer under a light microscope (Olympus Co, Japan). Oocysts were counted in four replicate chambers per sample.

### RNA isolation, cDNA synthesis, and qRT-PCR

Total RNA was extracted from spleen and cecum samples using the TRIzol Reagent Kit (Invitrogen, City, State, USA) according to the manufacturer’s instructions. The RNA quality was assessed using a NanoDrop 2000 spectrophotometer (Thermo Fisher Scientific, Inc., Waltham, MA, USA). RNA purity was evaluated on the basis of absorbance (A) ratios at specific wavelengths: 2.0˃A_260_/A_280_˃1.8, and A_260_/A_230_ ˃ 2.0 were considered to indicate high purity. RNA integrity was determined by electrophoretic analysis of the 28S:18S ribosomal RNA (rRNA) ratio.

Two micrograms of total RNA was treated with RNase-free DNase and reverse-transcribed according to manufacturer’s instructions (Takara Bio, Dalian, China). For quantitative PCR (qPCR), 2 μL of diluted complementary DNA (cDNA) (1:10, vol/vol) was used. Glyceraldehyde 3-phosphate dehydrogenase (GAPDH) was used as the reference gene, as its expression level remained relatively stable across all time points and experimental groups. Reactions were performed on a CFX Connect Real-Time PCR System (Bio-Rad, City, State, USA). Specific primer sequences are presented in Table 1. The PCR protocol consisted of an initial denaturation at 95 ℃ for 5 min, followed by 40 cycles of denaturation at 95 ℃ for 15 s, annealing at temperatures specific to each primer set (Table 1), and extension at 72 ℃ for 30 s. Each reaction included a no-template control to monitor potential contamination.

**Table 1:** Primer pair sequences used in this study

Gene	Forward primer and reverse primer (5′ → 3′)	Annealing temperature (°C)	Accession no.
IFN-γ	AGCTGACGGTGGACCTATTATT GGCTTTGCGCTGGATTC	55	NM_205149.2
IL-1β	TGGGCATCAAGGGCTACA TCGGGTTGGTTGGTGATG	56	NM_204524.2
TNF-α	TGTGTATGTGCAGCAACCCGTAGT GGCATTGCAATTTGGACAGAAGT	58	AY765397.1
IL-17	AAGCGGTTGTGGTCCTCAT CTCCGATCCCTTATTCTCCTC	55	NM_204460.2
iNOS	TGGGTGGAAGCCGAAATA GTACCAGCCGTTGAAAGGAC	55	NM_204961.1
ChTLR1a	TTACTGCCAATTGCTTGCAC GGTTAGGAAGACCGTGTCCA	56	AY633574
ChTLR1b	CCCGTTCAAGTGTTCATGTG GTTCCGCTCAAGTCTTCTGG	57	NM001081709
ChTLR2a	ACATGTGTGAATGGCCTGAA TTGAGAAATGGCAGTTGCAG	56	NM204278
ChTLR2b	TTCGCTCCAACACCTTCG CTGATGACTGCTGAAATACG	58	NM001011691
TLR3	AAGCCGCAGCATAGCATCATACC CCAGAGGAGTGAGTGAGGTGACAG	59	JF273967.1
TLR4	CATCCCAACCCAACCACAGTAGC CCACTGAGCAGCACCAATGAGTAG	61	XM_042879007.1
TLR5	ACTCCCTTCCTTCCCACATCTGAC TGTGTTGCTACTATTGCCGTGTGAG	58	NM_001398059.1
TLR7	TCTGGCTGTGAATGAATGGGTGATG GTCAAAGACTGGCTGTCCTGGAAG	58	NM_001011688.3
TLR15	GATGGGCTGTGGTATGTGAGAATGG TCAGTAGATGCTCCTTCGTCCAGTC	59	MH143572.1
TLR21	TCTCACAGGCGGAGGTCTTCAC GCGAGGTTGGATGTCAGAGATGTC	61	NM_001030558.3
MyD88	TGATGCCTTCATCTGCTACTG TCCCTCCGACACCTTCTTTCTA	56	OM291731.1
NF-κBp65	CAGCCCATCTATGACAACCG TCAGCCCAGAAACGAACCTC	57	D13721.1
GAPDH	TGCTGCCCAGAACATCATCC ACGGCAGGTCAGGTCAACAA	59	K01458

Relative expression levels were calculated using 2^*−*ΔΔCt^ method [17]. For each time point, the ΔCt value was calculated as Ct (target gene)-Ct (GAPDH). The ΔΔCt value was then calculated as ΔCt (infected)-ΔCt (time-matched uninfected control), where the time-matched uninfected control (sampled at the same time point from the mock-infected group) served as the calibrator. The fold change for each infected sample was expressed as 2^*−*ΔΔCt^ relative to the time-matched uninfected control (set to 1 at each time point). Uninfected control groups were sampled independently at every time point (0–168 hpi) to account for age-related changes in gene expression. Each quantitative reverse transcription polymerase chain reaction (qRT-PCR) reaction was performed in triplicate for each sample. The mean Ct value of the three technical replicates was used for subsequent analysis. Five independent biological replicates (*n* = 5 per group per time point) were included in the study.

### Statistical analysis

All statistical analyses were performed using GraphPad Prism 8 (GraphPad Software, San Diego, CA, USA). Data are presented as mean ± standard deviation (SD). Normality of the distribution of tissue immune gene expression levels and oocyst counts within each experimental group was assessed using the Shapiro–Wilk test (all *P* > 0.05), supported by visual inspection of normality plots. Homogeneity of variances across groups was verified using Brown-Forsythe test (*P* > 0.05). Subsequently, significant differences among groups were evaluated using one‑way analysis of variance (ANOVA), followed, where appropriate, by Tukey’s honestly significant difference (HSD) post‑hoc test for multiple comparisons. Differences were considered statistically significant at *P* < 0.05. Spearman’s rank correlation analysis was performed using the correlation matrix function in GraphPad Prism 8 to assess relationships between gene expression levels and pathogenicity parameters (oocyst counts and lesion scores). Correlation results are presented as heatmaps.

## Results

### Clinical signs, lesion scoring, and mortality rate

**Fig. 1:**
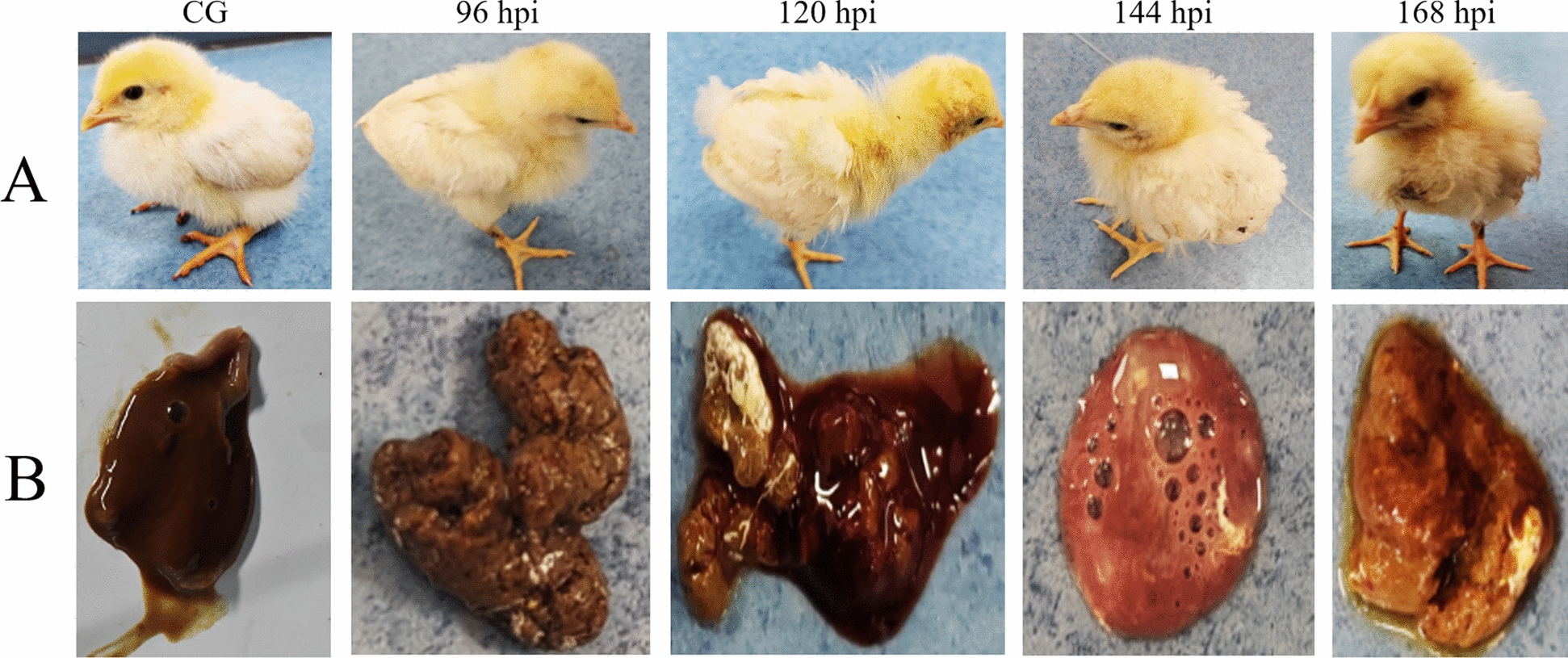
Representative images of clinical signs from *E. tenella*-infected and control chickens at 96–168 hpi. SPF chickens (14 days old) were orally infected with 5 × 10^4^ sporulated *E. tenella* oocysts (infected group) or PBS (control group). At each time point (96, 120, 144, and 168 hpi), five chickens per group were examined. Images shown are representative of five independent birds per group per time point. Clinical signs images of chickens postinfection of challenge. **A** Infected birds with marked clinical signs; **B** Bloody feces in infected chickens. *CG* control group

**Fig. 2:**
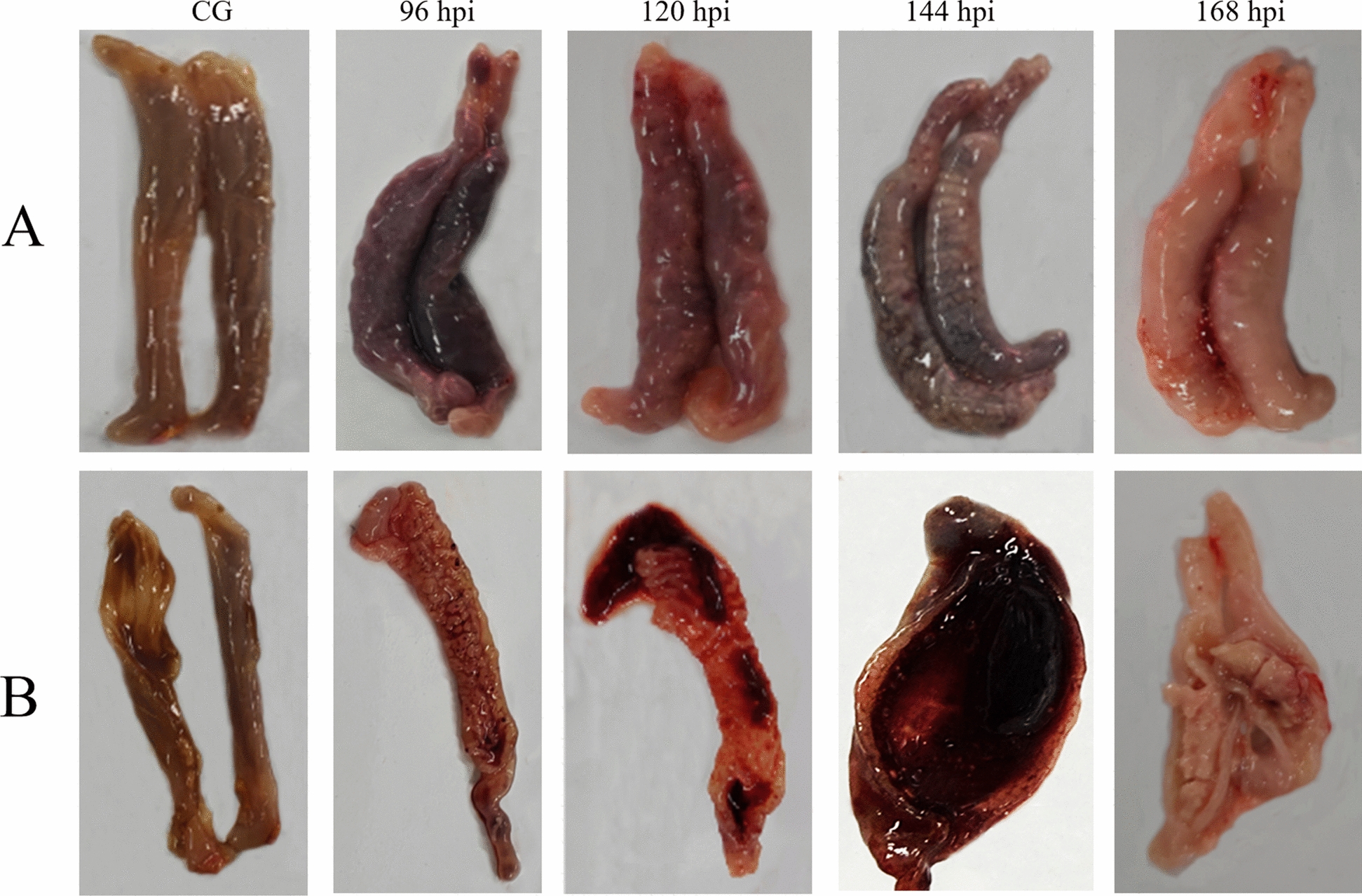
Representative images of Cecal lesions from *E. tenella*-infected and control chickens at 96–168 hpi. SPF chickens (14 days old) were orally infected with 5 × 10^4^ sporulated *E. tenella* oocysts (infected group) or PBS (control group). At each time point (96, 120, 144, and 168 hpi), five chickens per group were examined. Images shown are representative of five independent birds per group per time point. Clinical signs images of chickens postinfection of challenge. **A** Ceca filled with hemorrhagic contents; **B** Ceca mucosal surface with pathological alterations. *CG* Control group

In this study, no mortality was observed in either the *E. tenella*-infected or control groups of chickens. As shown in Fig. 1, At 96 hpi, infected chickens exhibited depression, lethargy, ruffled feathers, and blood in the feces. At 120 hpi, clinical signs progressed to marked lethargy, huddling behavior, rough feathers, and increased bloody diarrhea. At 144 hpi and 168 hpi, clinical signs persisted, with fecal soiling of the feathers around the anus. Specifically, at 144 hpi, chickens showed watery bloody diarrhea, whereas at 168 hpi, soft bloody feces were observed. Upon necropsy, cecal lesions progressed over time as shown in Fig. 2. At 96 hpi, moderate cecal enlargement and mucosal petechial hemorrhages were observed. At 120 hpi, severe changes were evident, including extensive mucosal hemorrhage and bloody contents. At 144 hpi, white striations and cecal cores appeared. At 168 hpi, cecal atrophy and reduced hemorrhage were observed. The average cecal lesion score was 2.5 at 96 hpi, peaking at 3.5 at 144 hpi (Table 2). Unchallenged control chickens displayed no lesions and received a score of zero. Although body weight was monitored throughout the experiment, no significant differences in body weight gain were observed between the *E. tenella*-infected and control groups at any time point (data not shown).

**Table 2:** Oocyst shedding in cecal contents of *E. tenella*-infected chickens at different time points post-infection

Hours post-infection (hpi)	3–72 hpi	96 hpi	120 hpi	144 hpi	168 hpi
Lesion score	–	2.5^*^ ± 0.11	3.3 ± 0.10	3.5 ± 0.09	3.0 ± 0.13
Number of oocytes shedding	–	–	4800^*^ ± 500	319,000^**^ ± 33,025	438,000^**^ ± 22,057

Significance thresholds were set at *P* < 0.05 (*) and *P* < 0.01 (**) for comparision with the controls

### Histopathological examination

**Fig. 3:**
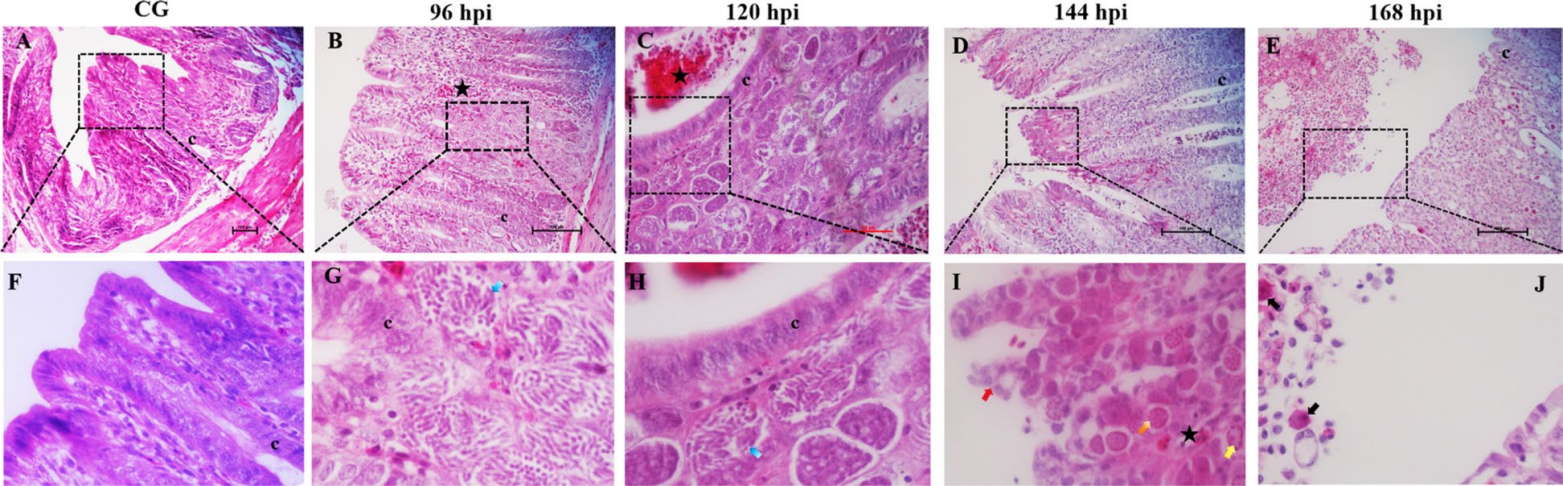
Representative images of Cecal morphology from *E. tenella*-infected and control chickens at 96–168 hpi. SPF chickens (14 days old) were orally infected with 5 × 10^4^ sporulated *E. tenella* oocysts (infected group) or PBS (control group). At each time point (96, 120, 144, and 168 hpi), five chickens per group were examined. Images shown are representative of five independent birds per group per time point. Arrows mark parasites, infiltration, and necrosis (200 × /400 ×). Scale bar: 100 µm. *CG* Control group

Histopathological examination of the ceca revealed marked differences between *E. tenella*-infected and control chickens, as shown in Fig. 3. Tissues from the control group exhibited normal structure, with no pathological alterations or parasites detected during under a microscopic examination. By contrast, infected birds developed progressive and severe cecal damage beginning at 96 hpi. Extensive colonization of cecal crypts by *E. tenella* resulted in severe architectural disruption and hyperplastic changes in the affected glands. Prominent heterophil infiltration was observed across multiple mucosal layers from 96 to 168 hpi, with particularly dense accumulations in the submucosa at 120 hpi and 144 hpi. Additional pathological features included necrosis, hemorrhagic changes, and sloughing of the mucosal epithelium from 96 to 120 hpi. Furthermore, various developmental stages of the parasite—including schizonts, gametocytes, and oocysts—were observed within enterocytes and the lamina propria. Numerous schizonts were present at 96 hpi, and by 168 hpi, oocysts were detected in the intestinal lumen.

**Fig. 4:**
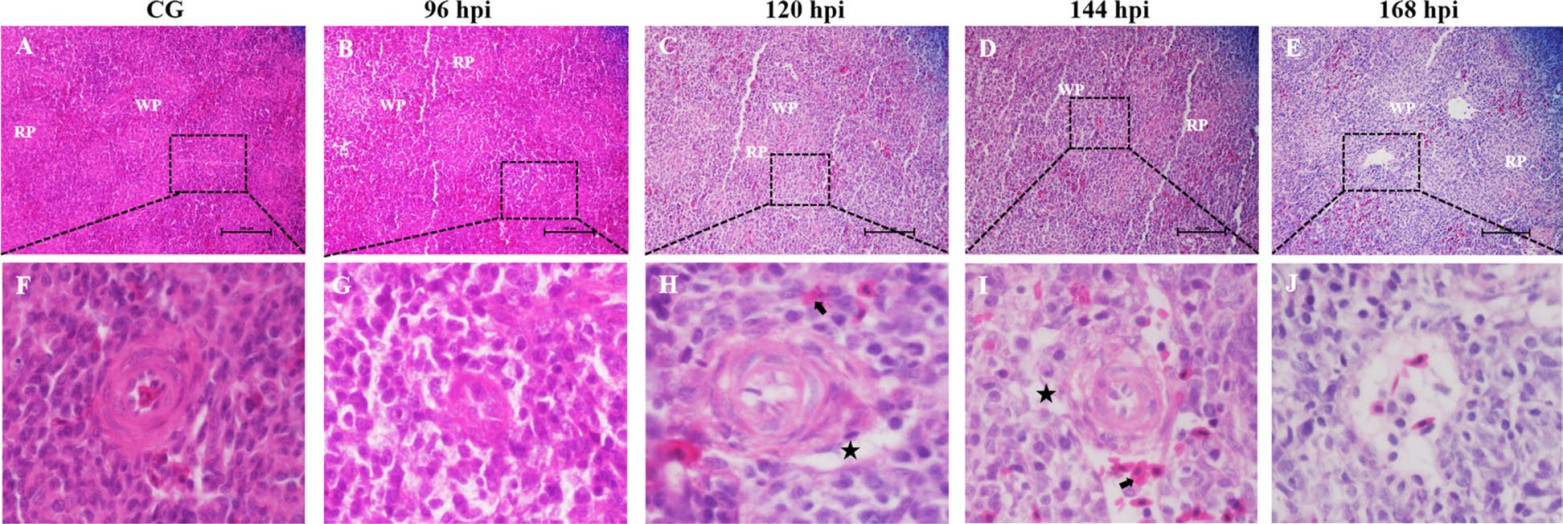
Representative images of splenic histopathology from *E. tenella*-infected and control chickens at 96–168 hpi. SPF chickens (14 days old) were orally infected with 5 × 10^4^ sporulated *E. tenella* oocysts (infected group) or PBS (control group). At each time point (96, 120, 144, and 168 hpi), five chickens per group were examined. Images shown are representative of five independent birds per group per time point. Arrows: inflammation; pentagrams: connective tissue hyperplasia (**A**–**J**, 200 × /400 ×). Scale bar: 100 µm. *CG* Control group

Concurrently, a series of progressive pathological changes were observed in the spleen tissues of infected birds (Fig. 4). From 3 to 72 hpi, no significant alterations were detected in the splenic corpuscles. By 96 hpi, however, notable changes emerged, characterized by mild structural deformation of both the white and red pulp, along with a progressive reduction in cellularity of the red pulp toward the end of the observation period. Between 96 and 144 hpi, connective tissue hyperplasia, inflammatory cell infiltration, and a decrease in lymphocyte numbers were observed in the periarterial lymphatic sheath (PALS) of the white pulp. Furthermore, At 168 hpi, the splenic tissue of infected chickens showed focal parenchymal loss, appearing as an empty space within the otherwise cellular tissue. By contrast, splenic tissues from the control group showed a tightly compact structure, a clear boundary between white and red pulp, and well-organized splenic cells.

In addition, the results showed that at 36 hpi and 72 hpi, no significant histopathological changes were observed in either the cecum or the spleen (Supplementary Fig. ?).

### Count of oocysts

Oocysts were first detected in cecal contents at 120 hpi, with a count of 4.8 × 10^3^, which increased rapidly to 3.19 × 10^6^ by 144 hpi and continued to rise in subsequent observation periods (Table 2).

### Expression of chTLRs mRNA in *E. tenella*-infected chickens

**Fig. 5:**
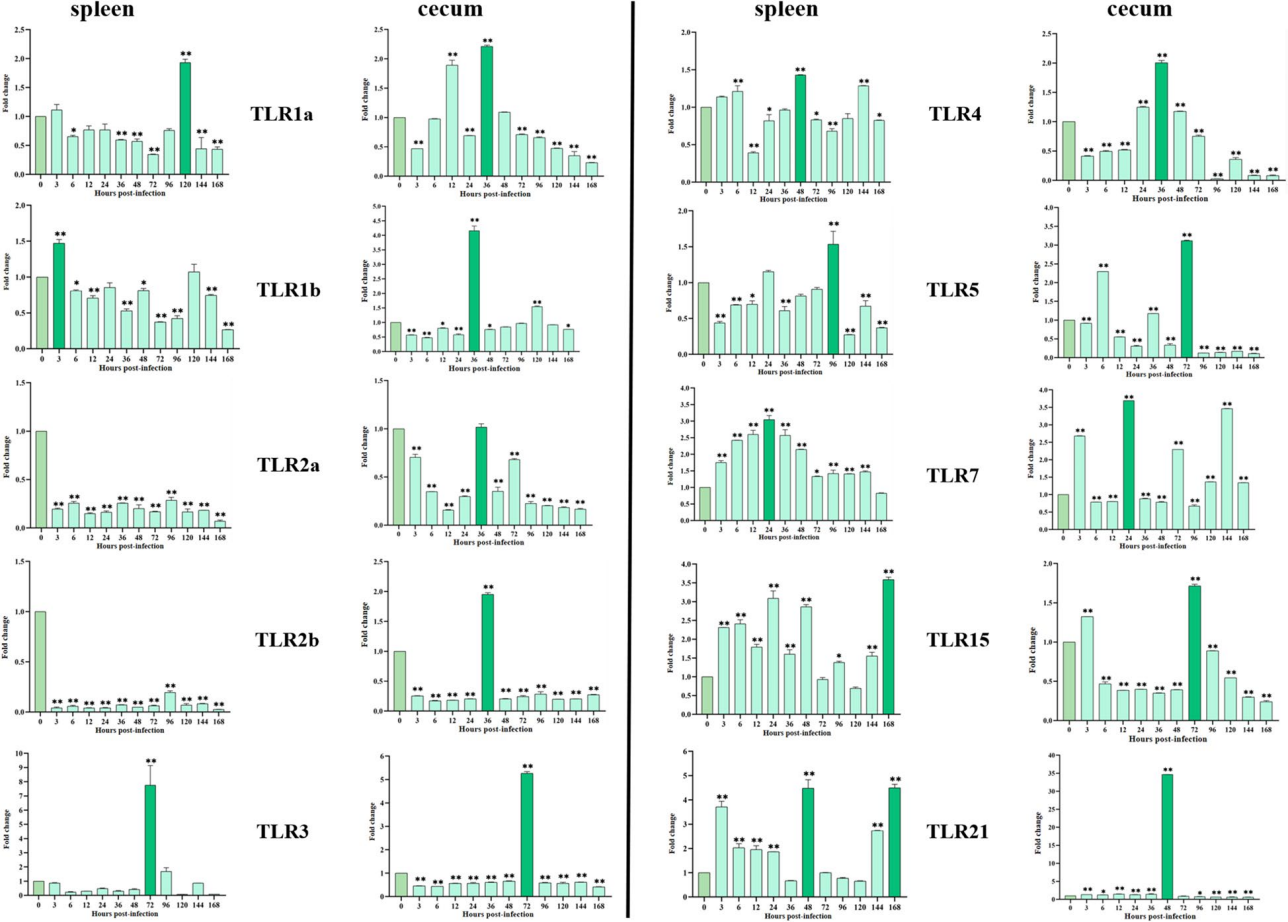
Expression changes of chTLR genes in the cecum and spleen of *E. tenella*-infected chickens. SPF chickens (14 days old) were orally infected with 5 × 10^4^ sporulated *E. tenella* oocysts (infected group) or PBS (control group). At each of the indicated time points (0, 3, 6, 12, 24, 36, 48, 72, 96, 120, 144, and 168 hpi), five chickens from each group were euthanized (*n* = 5 per group per time point). Cecum and spleen tissue were collected for RNA extraction. Gene expression was quantified by qRT-PCR using GAPDH as an internal reference. Fold changes were calculated using the 2^⁻ΔΔCt^ method relative to time-matched uninfected controls (set to 1 at each time point). Data are presented as mean ± SEM. *P < 0.05, **P < 0.01 compared with time-matched controls (one-way ANOVA followed by Tukey’s post-hoc test)

To elucidate the innate immune response triggered by *E. tenella* infection, we analyzed the expression profiles of chTLRs in the cecum and spleen at twelve time points using qRT–PCR. The relative expression of each target gene was determined by comparing infected groups with the uninfected control. As illustrated in Fig. 5, distinct expression patterns were observed across different chTLRs and tissues. In the cecum, almost all chTLRs upregulation at mid-phase (24–72 hpi) of infection. Among these, chTLR1a, chTLR1b, chTLR2b, and chTLR4 were upregulated at 36 hpi (*P* < 0.01), with TLR1b showing the highest fold change of of 4.2. Upregulation of TLR3, TLR5, and TLR15 was observed at 72 hpi (*P* < 0.01), with chTLR3 expression (5.2-fold) being higher than that of chTLR5 and chTLR15. By contrast, chTLR2a expression was markerly lower than that of the control group. In the spleen, TLR1b was elevated as early as 3 hpi (1.4-fold, *P* < 0.01), followed by TLR1a at 120 hpi (2.0-fold, *P* < 0.01) and TLR4 at 48 hpi (1.45 fold, *P* < 0.01). TLR5 upregulation occurred at 96 hpi (1.5-fold), slightly later than that in the cecum (3.1-fold in cecum), while TLR15 upregulation was delayed until 168 hpi (3.6-fold). By contrast, both TLR2a and TLR2b were significantly downregulated in the spleen from the initial infection and continued to decrease throughout the observation period (*P* < 0.01).

Notably, several TLRs were upregulated in both tissues, though with varying kinetics. TLR7 showed increased expression in both the cecum and spleen at 24 hpi (both ˃3.0-fold), while TLR3 was significantly upregulated in both organs at 72 hpi, with fold change of 7.5 and 5.4, respectively (*P* < 0.01). TLR21 was upregulated in both tissues at 48 hpi, reaching 35-fold in the cecum, and exhibited a second significant upregulation in the spleen at 168 hpi (4.5-fold). Among all TLRs analyzed, TLR21 showed the highest expression level, with a 35-fold increase in the cecum.

### Expression of cytokines and other immunity-related genes in response to *E. tenella* infection

**Fig. 6:**
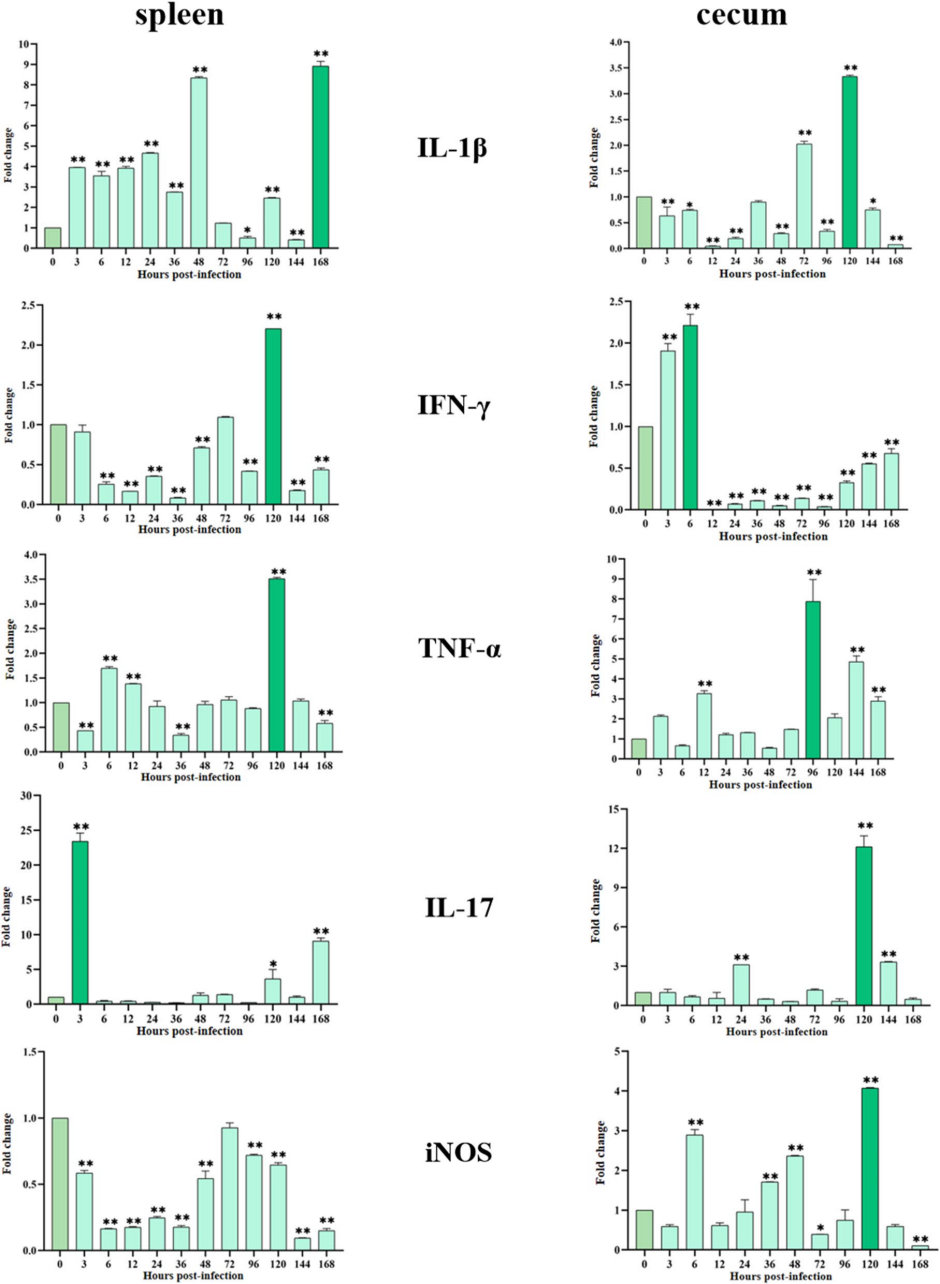
Expression changes of cytokine genes in the cecum and spleen of *E. tenella*-infected chickens. SPF chickens (14 days old) were orally infected with 5 × 10^4^ sporulated *E. tenella* oocysts (infected group) or PBS (control group). At each of the indicated time points (0, 3, 6, 12, 24, 36, 48, 72, 96, 120, 144, and 168 hpi), five chickens from each group were euthanized (*n* = 5 per group per time point). Cecum and spleen tissue were collected for RNA extraction. Gene expression was quantified by qRT-PCR using GAPDH as an internal reference. Fold changes were calculated using the 2^⁻ΔΔCt^ method relative to time-matched uninfected controls (set to 1 at each time point). Data are presented as mean ± SEM. *P < 0.05, **P < 0.01 compared with time-matched controls (one-way ANOVA followed by Tukey’s post-hoc test)

The mRNA expression levels of several cytokines—including interferon‑γ (IFN‑γ), interleukin (IL)‑1β, IL‑17, tumor necrosis factor (TNF)‑α, and inducible nitric oxide synthase (iNOS)—were assessed in the cecum and spleen of infected and control chickens following *E. tenella* infection (Fig. 6). In the cecum, IFN‑γ expression increased significantly at 3 hpi (1.9‑fold, *P* < 0.01), peaked at 6 hpi (2.2‑fold, *P* < 0.01), and then declined throughout the remainder of the experiment. By contrast, splenic IFN‑γ expression initially decreased, followed by a significant peak at 120 hpi (2.3‑fold, *P* < 0.01). IL‑17 was markedly upregulated in the spleen as early as 3 hpi (24‑fold, *P* < 0.01), whereas its upregulation in the cecum occurred later, at 120 hpi (12‑fold, *P* < 0.01). A delayed response was observed for other cytokines. Cecal IL‑1β expression increased significantly at 72 hpi (2.0‑fold, *P* < 0.01) and peaked at 120 hpi (3.4‑fold, *P* < 0.01). In the spleen, IL‑1β expression was elevated at 3 hpi (3.9‑fold, *P* < 0.01), increased further at 48 hpi (8.1‑fold, *P* < 0.01), and reached its peak at 168 hpi (8.9‑fold, *P* < 0.01). TNF‑α peaked in the cecum at 96 hpi (7.9‑fold, *P* < 0.01), followed by an increase at 144 hpi (5.0‑fold, *P* < 0.01). Splenic TNF‑α was significantly elevated at 6 hpi (1.6‑fold, *P* < 0.01) and peaked at 120 hpi (3.5‑fold, *P* < 0.01). Notably, iNOS expression was significantly suppressed in the spleen throughout the entire experimental period. Conversely, it was transiently upregulated in the cecum, showing a 4.2-fold increase at 120 hpi, and marked upregulation was also observed at 6 hpi (2.9-fold, *P* < 0.01), 36 hpi (1.8-fold, *P* < 0.01), and 48 hpi (2.5-fold, *P* < 0.01).

**Fig. 7:**
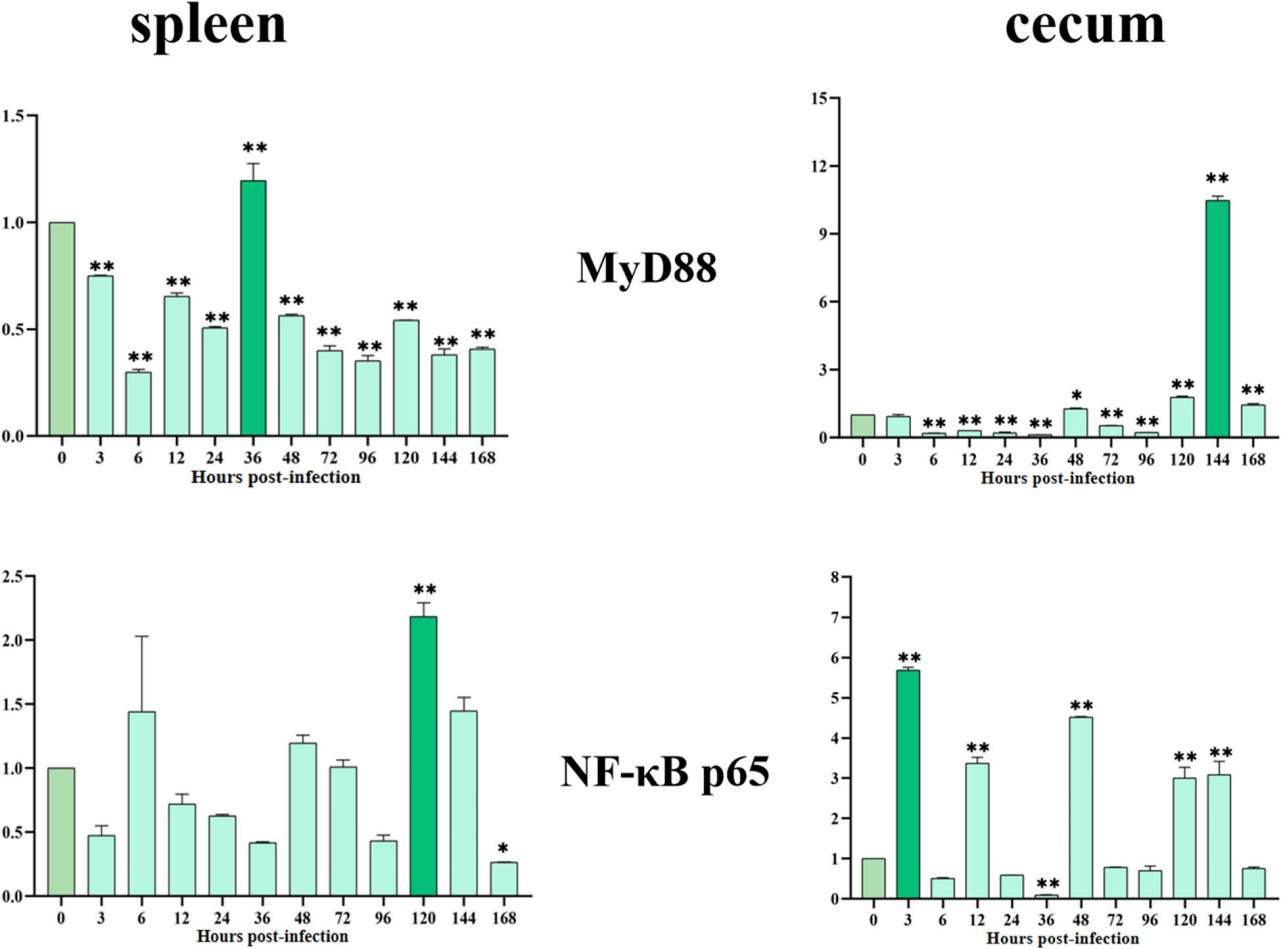
Expression changes of transcription molecules genes in in the cecum and spleen of *E. tenella*-infected chickens. SPF chickens (14 days old) were orally infected with 5 × 10^4^ sporulated *E. tenella* oocysts (infected group) or PBS (control group). At each of the indicated time points (0, 3, 6, 12, 24, 36, 48, 72, 96, 120, 144, and 168 hpi), five chickens from each group were euthanized (*n* = 5 per group per time point). Cecum and spleen tissue were collected for RNA extraction. Gene expression was quantified by qRT-PCR using GAPDH as an internal reference. Fold changes were calculated using the 2^⁻ΔΔCt^ method relative to time-matched uninfected controls (set to 1 at each time point). Data are presented as mean ± SEM. *P < 0.05, **P < 0.01 compared with time-matched controls (one-way ANOVA followed by Tukey’s post-hoc test)

The mRNA expression levels of key innate immune signaling components, including the adaptor protein myeloid differentiation factor 88 (MyD88) and nuclear factor-kappa B (NF-κB), were analyzed in the cecum and spleen of *E. tenella*-infected and control chickens. As shown in Fig. 7, distinct spatiotemporal expression patterns were observed for both genes. In the cecum, MyD88 was significantly upregulated at 144 hpi (11.5-fold). By contrast, in the spleen, its elevation occurred much earlier, at 36 hpi (1.2-fold, *P* < 0.01), followed by significant downregulation that persisited until the end of study, with fold changes consistently below those of the control group. NF-κB expression was significantly increased in the cecum as early as 3 hpi (5.8-fold, *P* < 0.01). By contrast, splenic NF-κB expression was initially downregulated at 3 hpi but showed significant upregulation by 120 hpi (2.2-fold, *P* < 0.01). These results collectively indicate that *E. tenella* infection activated TLR-mediated pathways, leading to the induction of proinflammatory cytokines.

### Correlations between parameters

**Fig. 8:**
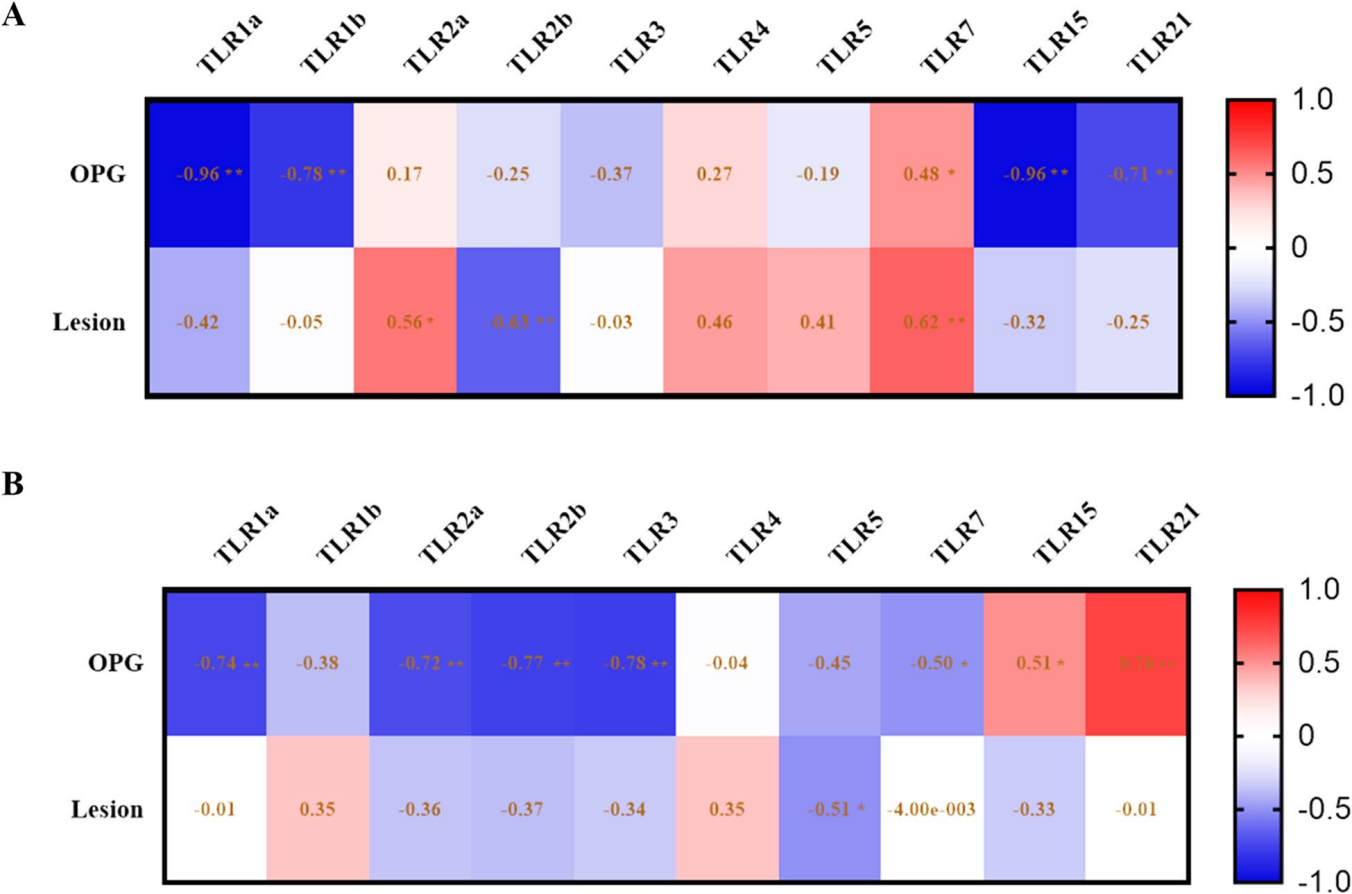
Spearman correlation heatmap between TLRs expression and pathogenicity indicators in cecum (**A**) and spleen (**B**). Spearman’s rank correlation coefficients (*r*) were calculated using GraphPad Prism 8. OPG: oocysts per gram of cecal content; lesion score (0–4 scale). Colors represent correlation strength: red indicates positive correlation, blue indicates negative correlation. *P < 0.05, **P < 0.01. Data were pooled from 96 to 168 hpi with *n* = 5 per group per time point

Spearman correlation heatmap revealed distinct correlation patterns between chTLR expression and pathogenicity parameters in the cecum and spleen (Fig. 8). In the cecum, chTLR7 showed strong positive correlations with both OPG (*r* = 0.48, *P* < 0.05) and lesion scores (*r* = 0.62, *P* < 0.01), and chTLR2a exhibited a strong positive correlation with lesion scores (*r* = 0.56, *P* < 0.05). By contrast, several chTLRs, including chTLR1a, chTLR1b, chTLR15, and chTLR21, displayed strong negative correlations with OPG (*r* values ranging from −0.96 to −0.71, all *p* < 0.01). Additionally, chTLR2b displayed strong negative correlations with Lesion scores (*r* = −0.63, *P* < 0.01).

In the spleen, chTLR15 and chTLR21 showed strong positive correlations with OPG (TLR15: *r* = 0.51, *P* < 0.05; chTLR21: *r* = 0.76, *P* < 0.01). By contrast, a cluster of TLRs (including chTLR1a, chTLR2a, chTLR2b, chTLR3, and chTLR7) exhibited strong negative correlations with OPG (*r* values ranging from −0.78 to −0.50). All correlations were significant, with chTLR7 at *P* < 0.05 and the remaining chTLRs at *P* < 0.01. Regarding lesion scores, chTLR5 was the only chTLR that showed a significant negative correlation (*r* = −0.51, *P* < 0.05); no other chTLRs exhibited significant correlations with this parameter.

## Discussion

*Eimeria tenella,* a highly pathogenic species of chicken coccidia, causes substantial economic losses to the global poultry industry. It primarily infects the epithelial cells of the cecal crypts, leading to clinical signs such as bloody diarrhea, weight loss, and in severe cases, mortality [18]. Toll-like receptors (TLRs), as key components of the innate immune system, have been shown to play a critical role in pathogen control and clearance [19]. Nevertheless, the function of TLR signaling in chickens during *E. tenella* infection remains incompletely elucidated. In the present study, the pathogenicity of *E. tenella* in 2-week-old chickens was evaluated through clinical assessment, gene expression analysis, and histopathological examination. The results demonstrate that chTLR genes are actively involved in host defense against *E. tenella* infection and provide a comprehensive overview of chTLR signaling during infection. Although no mortality or significant body weight loss was observed following infection, pathological changes—including cecal lesions, oocyst shedding, and inflammatory infiltration—were confirmed. On the basis of these collective findings, the infection model used in this study may be characterized as a subclinical infection model. This subclinical model allows investigation of host innate immune responses without the confounding effects of severe morbidity or mortality, underscoring the essential role of chTLR signaling in mediating acute inflammatory responses in *Eimeria*-affected chickens.

In the present study, a dose of 5 × 10^4^ sporulated *E. tenella* oocysts per chicken was used. On the basis of previous reports, this dose is considered moderate-to-severe and sublethal in SPF chickens [20, 21]. Previous studies have shown that the choice of challenge dose depends on the experimental objective: low doses (100–250 oocysts) are used to evaluate the efficacy of novel vaccines, whereas high doses (5000–50,000 oocysts) are used to assess disease pathology [22]. In the current study, no mortality was observed following infection with 5 × 10^4^ oocysts, which differs from Sun et al., who reported a 40% mortality rate at the same dose and 100% mortality at 5 × 10^5^ oocysts [18]. Nevertheless, significant pathogenic effects were confirmed in this investigation, as demonstrated by substantial oocyst shedding in cecal contents from 120 to 168 hpi, variable degrees of cecal lesions, and persistent inflammatory cell infiltration in the cecum throughout the experimental period. These results suggest that, even for highly pathogenic *Eimeria* species, mortality is not an inevitable outcome. Instead, the outcome of infection is shaped by a complex interplay of factors, including the inherent virulence of the parasite strain, the infection dose, and host-related variables such as age, breed, and genetic background [23]. For *E. tenella*, studies have shown that mortality is dose-dependent: doses of 5 × 10^4^ oocysts can induce significant pathological effects without causing death [24], whereas higher doses (≥ 5 × 10^5^ oocysts) result in 100% mortality [18]. Therefore, the selected dose of 5 × 10^4^ oocysts was optimal for the current experimental design, which required all birds to survive until the final time point (168 hpi) to allow complete characterization of innate immune responses.

*Eimeria* spp., the causative agents of poultry coccidiosis, trigger significant pathological alterations in the cecum of infected birds, predominantly characterized by mucosal damage and inflammatory cell infiltration [25]. In the present study, histopathological analysis of infected cecal samples revealed substantial heterophil infiltration in the mucosal and submucosal layers, along with sloughing of intestinal lining cells. These findings are consistent with previous reports describing massive heterophil recruitment into the cecal mucosa during the mid to late stages of *Eimeria* infection, a response associated with the release of antimicrobial peptides that help control parasite replication [21]. In addition, parasite invasion of the intestinal epithelium was associated with extensive apoptosis of cecal epithelial cells and shedding of the mucosal layer, which, combined with inflammatory cell infiltration, collectively contributing to severe mucosal injury. Various developmental stages of the parasite were observed within infected epithelial cells, corroborating previous studies identifying the second-generation schizont as the most pathogenic stage of *E. tenella*, known to cause extensive cecal hemorrhage and loss of tissue integrity [26]. Consistent with these reports, the current study detected numerous second-generation schizonts at 96 hpi and 120 hpi. Located deep within the lamina propria, the maturation of these schizonts and subsequent release of merozoites led to vascular rupture and substantial tissue destruction.

In chickens, the spleen is a major peripheral immune organ, where the white pulp functions as the primary site for antigen‑specific immune responses and supports T‑lymphocyte proliferation [27]. In the present study, a reduction of red pulp and a marked decline in lymphocyte numbers in the white pulp were observed following *E. tenella* infection. These progressive structural alterations likely reflect the extensive parasitic invasion in the cecum—the specific target tissue—which triggers the release of pro‑inflammatory cytokines. These cytokines enter the bloodstream through the damaged cecal mucosa, subsequently inducing lymphocyte apoptosis, inflammatory cell infiltration, and connective tissue hyperplasia. Together, these processes disrupt normal tissue architecture, ultimately leading to focal parenchymal loss that appears as empty spaces within the otherwise cellular tissue.

The innate immune system initiates host defense by recognizing pathogen-associated molecular patterns (PAMPs), subsequently orchestrating inflammatory responses and the secretion of proinflammatory cytokines to eliminate pathogens [28]. In the present study, we observed a significant upregulation of several immune-related genes, including chTLR1b, IFN-γ, and IL-17, during the early stage of infection. By contrast, the expression of chTLR3, chTLR4, chTLR7, and chTLR21 increased during the mid-phase (24–72 hpi) of infection. Previous studies have suggested that inducing the expression of chTLRs and specific cytokines can enhance antiparasitic immunity in birds [29, 30]. Thus, the elevated expression of chTLRs and cytokines observed in the current study likely contributes to the protective immunity developed in the tissues of infected birds.

In the present study, chTLR1b was upregulated in the spleen during the early stage (3–12 hpi), in the cecum during the mid-phase (24–72 hpi), whereas chTLR2a was unchanged in the cecum and downregulated in the spleen. Although chTLR2a forms functional heterodimers with chTLR1b [31], this discordant expression suggests alternative possibilities: chTLR2a may dimerize with other partners (e.g., chTLR2b) [32]; chTLR2a may be constitutively expressed at sufficient levels [33]; or post-transcriptional regulation may control its activity [34]. Further studies are needed to clarify the functional implications of these discordant patterns. The expression of chTLR3, chTLR4, chTLR7, and chTLR21 was upregulated in both the cecum and spleen during the mid-stage (24–72 hpi) of infection. Although chTLR3 and chTLR7 are known for their antiviral functions—with altered expression reported during IBDV infection [35]—their upregulation here indicates a distinct role in *E. tenella* infection. Notably, chTLR21 exhibited the most pronounced upregulation among all selected chTLRs in the cecum. Tissue-and pathogen-specific regulation of chTLR21 has been documented: it is expressed primarily in immune-related tissues and shows differential expression during infections such as fowl adenovirus [7, 36], underscoring that its mRNA expression varies with pathogen type and infection context. chTLR4, which recognizes Gram-negative bacterial LPS [37], has been shown to increase during various parasitic infections [38–40]. Its upregulation in the present study aligns with these findings, though its timing in the cecum and spleen appears earlier than that reported for *E. praecox* in the duodenum and jejunum [41]. Interestingly, the downregulation of chTLR2a and chTLR2b wsas observed alongside the upregulation of chTLR4, suggesting contrasting roles that may reveal potential therapeutic targets for coccidiosis control. The underlying molecular mechanism is likely complex, involving multiple signaling cascades. Finally, chTLR5 and chTLR15 displayed distinct tissue-specific kinetic profiles. In the cecum, chTLR5 was significantly upregulated at 6 hpi, 36 hpi, and 72 hpi, and chTLR15 was upregulated at 3 hpi and 72 hpi, indicating an early local response at the primary site of infection. By contrast, splenic chTLR5 upregulation occurred later (96 hpi), and splenic chTLR15 upregulation was delayed until 168 hpi, suggesting that systemic TLR responses in the spleen are activated after the local cecal response. This temporal difference may reflect the sequential activation of innate immunity from the local site of infection to systemic immune organs. Overall, our results demonstrate that chTLR1b, chTLR3, chTLR4, chTLR7, chTLR21, chTLR5, and chTLR15 are involved in parasite recognition at different timepoints and contribute to the host defense against *E. tenella* infection.

Upon activation by parasite-derived ligands, chTLRs trigger downstream signaling cascades that induce the expression of a wide range of cytokines, including key proinflammatory mediators [29, 42]. As chickens lack lymph nodes, T lymphocyte proliferation occurs predominantly in the spleen. IFN-γ, IL-1β, and TNF-α, as critical proinflammatory cytokines, whose transcriptional levels are closely associated with parasite burden and clinical manifestations in infected birds [43, 44]. Similarly, expression changes occurred after infection with *E. tenella* in the present study. The results of the study showed that the expression of these three cytokines almost peaked at a later stage of infection (96–168 hpi) in the both cecum and spleen, with exception of upregulation of IFN-γ peaking at 6 hpi in the cecum. In the cecum, IFN-γ expression was increased rapidly at 3 hpi, peaking at 6 hpi before declining thereafter. By contrast, splenic IFN-γ showed initially decreased, followed by a significant increase at 120 hpi, indicating that *E. tenella* infection induced tissue-specific expression profiles of IFN-γ. Furthermore, several studies have shown that TNF-α can play a vital role in regulating host resistance to *Neospora caninum* and *T. gondii* infection [45, 46]. The expression of TNF-α in the current study was all peaked at later phase of infection (96–120 hpi), although they significantly upregulated at early stage of infection (cecal TNF-α at 3 hpi, spleenic TNF-α at 6 hpi). Similarly, the expression of IL-1β in the present study also peaked at later phase of infection (96–120 hpi), spleenic IL-1β rapidly increased at 3 h post infection, then continued to increase at 48 h post infection, and then peaked at 168 h post infection, while cecal IL-1β was markedly increased at 72 hpi, and peaked at 120 hpi, suggesting that TNF-α and IL-1β expression occurred not only at the middle stage but also at later stages during *E. tenella* infection.

In addition, the expression of IL-17 and inducible nitric oxide synthase (iNOS) was assessed to further elucidate the inflammatory response to *E. tenella* infection. The results revealed a marked increase in IL-17 expression in the spleen as early as 3 hpi, with additional significant upregulation at 120 hpi and 168 hpi. In the cecum, significant upregulation was first observed at 24 hpi, peaked at 120 hpi, and remained elevated at 144 hpi. This distinct spatiotemporal pattern implies that splenic IL-17 may be pivotal in both early and late systemic immune responses, whereas cecal IL-17 contributes to both delayed and sustained local immunity. Concurrently, iNOS—a key enzyme in antiparasitic defense that catalyzes the production of nitric oxide (NO), a molecule with direct inhibitory and parasiticidal effects—exhibited contrasting regulation: its expression was upregulated in the cecum during the later stage of infection but was significantly suppressed in the spleen throughout the experimental period [47]. This tissue-specific response suggests that *E. tenella* infection may selectively induce iNOS at the primary site of infection to facilitate local NO-mediated parasite clearance, while potentially repressing its expression in systemic immune organs such as the spleen. The precise mechanisms responsible for this differential regulation, however, warrant further investigation.

The results of this study revealed a distinct tissue-specific expression profile of key signaling molecules induced by *E. tenella* infection. In the spleen, MyD88 was markedly upregulated at 36 hpi, and an obvious increase in NF-κB p65 expression at 120 hpi. Conversely, in the cecum, NF-κB p65 expression rose sharply at 3 hpi, while MyD88 upregulation at 144 hpi. As the first identified adaptor protein of the TIR family, MyD88 is recruited by nearly all chTLRs except chTLR3. It activates the transcription factor NF-κB and mitogen-activated protein kinases, driving the production of inflammatory cytokines and thereby mediating parasite-induced inflammation [38]. On the basis of the observed expression dynamics, we speculate that in the spleen, MyD88 may function upstream to activate the NF-κB p65 pathway as part of the host defense against *E. tenella*. However, the precise molecular mechanisms governing this tissue-specific regulatory process require further investigation.

Spearman correlation analysis revealed tissue-specific associations between chTLR expression and pathogenicity parameters (OPG, lesion scores). In the cecum, chTLR7 correlated positively with both OPG and lesion scores, and chTLR2a correlated positively with lesion scores, suggesting these chTLRs may promote parasite replication and local tissue damage. By contrast, chTLR1a, chTLR1b, chTLR15, and chTLR21 showed strong negative correlations with OPG, possibly reflecting regulatory/anti-inflammatory pathways or host strategies to limit excessive inflammation. In the spleen, chTLR15 and chTLR21 correlated positively with OPG, indicating systemic chTLR responses are linked to intestinal parasite burden, whereas a cluster of TLRs (chTLR1a, chTLR2a, chTLR2b, chTLR3, and chTLR7) correlated negatively with OPG, suggesting tissue-specific suppression during high parasite loads. For lesion scores, only chTLR5 showed a negative correlation in the spleen, implying a protective or anti-inflammatory role. Notably, the correlations between TLR7 and TLR15 with OPG were opposite in the cecum and spleen. In the cecum, TLR7 showed a positive correlation with OPG, whereas in the spleen, it showed a negative correlation. Conversely, TLR15 was negatively correlated with OPG in the cecum but positively correlated in the spleen. Collectively, these results indicate that TLR-pathogenicity relationships are complex and tissue-specific, with positive correlations potentially reflecting proinflammatory roles and negative correlations suggesting regulatory feedback or protective mechanisms. Further research are required to determine the causal relationships suggested by these correlations.

The present study revealed that *E. tenella* infection induced distinct expression profiles of chTLRs, proinflammatory cytokines (IFN-γ, IL-1β, TNF-α) and key signaling molecules (MyD88, NF-κB p65). In the cecum, the positive correlations between expression of chTLR7/chTLR2a and lesion scores/OPG, and coupled with the concurrent upregulation of NF-κB p65 and proinflammatory cytokines, suggest a mechanistic link: TLR-mediated activation of the NF-κB pathway may drive the production of inflammatory mediators that contribute to mucosal necrosis, hemorrhage, tissue damage, and oocyst development. In the spleen, the expression pattern of IFN-γ was similar to that of NF-κB p65, suggesting a potential connection between IFN-γ production and the NF-κB signaling pathway in this tissue. Therefore, these findings indicate that the NF-κB pathway may play a central role in mediating proinflammatory responses in both local and systemic compartments during *E. tenella* infection.

Several limitations of this study should be acknowledged. First, the use of SPF chickens limits extrapolation to commercial flocks. Second, clinical assessment was qualitative and nonblinded, and feed intake was not measured, limiting quantitative clinical parameters. Third, only mRNA expression was analyzed; protein-level validation is needed to confirm the functional relevance of the observed transcriptional changes. Fourth, the study is correlational; functional validation (e.g., TLR knockdown/overexpression) is required to establish causality to determine whether these correlations reflect direct regulatory roles. Future studies should address these limitations by validating findings in commercial chickens, using standardized clinical scoring, performing protein-level analyses, and conducting in vitro functional experiments to confirm the roles of TLR signaling in host defense against coccidiosis.

## Conclusions

This study reveals correlations between chTLR expression and *E. tenella* pathogenicity. In the cecum, chTLR7 positively correlated with both oocyst output and lesion scores, whereas chTLR2a positively correlated with lesion scores. By contrast, chTLR1a, chTLR1b, chTLR15, and chTLR21 negatively correlated with oocyst output. In the spleen, chTLR15 and chTLR21 positively correlated with oocyst output, while chTLR5 uniquely and negatively correlated with lesion scores. These findings identify both proinflammatory and potentially protective chTLR pathways in subclinical coccidiosis. Future investigations are required to characterize the molecular mechanisms by which TLR signaling regulates proinflammatory and protective immune responses during *E. tenella* infection, and to establish causality through functional validation experiments.

## Supplementary Information

Supplementary material 1.

## Data Availability

Data supporting the main conclusions of this study are included in the manuscript.

## Abbreviations

PCR: Polymerase chain reaction
SPF: Specific pathogen free
qRT-PCR: Quantitative reverse transcription polymerase chain reaction
