# Supplementary material for: Toll-like receptor-mediated innate immune response correlate with the pathogenicity of Eimeria tenella infection in SPF chickens
Source: Parasit Vectors. 2026 May 27;19:299. doi: 10.1186/s13071-026-07466-2 (PMC13397759; doi:10.1186/s13071-026-07466-2)
Supplement: Supplementary file 1 — Supplementary material 1. [file 13071_2026_7466_MOESM1_ESM.pdf]

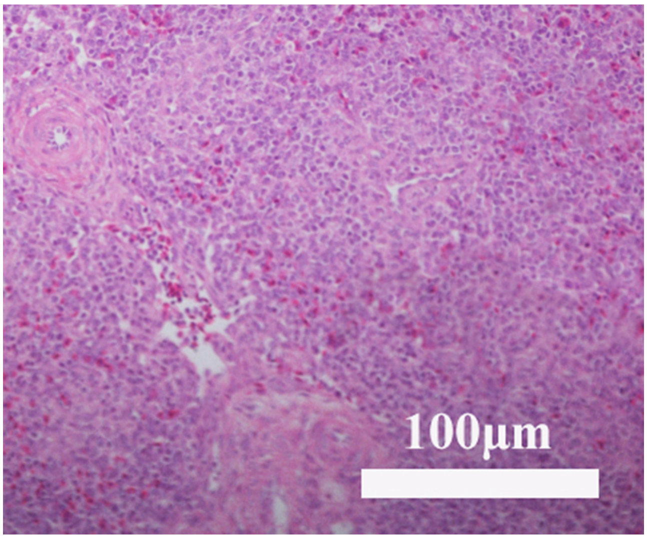

Fig. 1 Representative images of splenic histopathology from *E. tenella*-infected chicken at 36 hpi

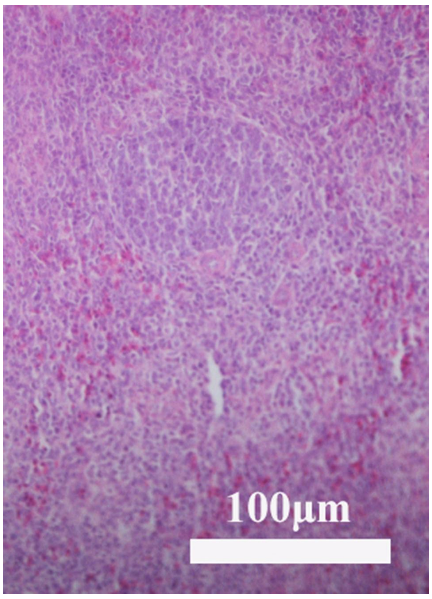

Fig. 2 Representative images of splenic histopathology from *E. tenella*-infected chicken at 72 hpi

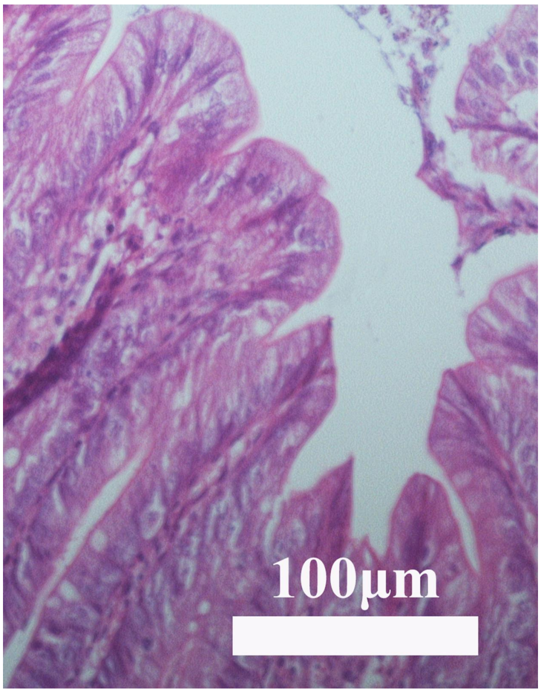

Fig. 3 Representative images of cecal histopathology from *E. tenella*-infected chicken at 36 hpi

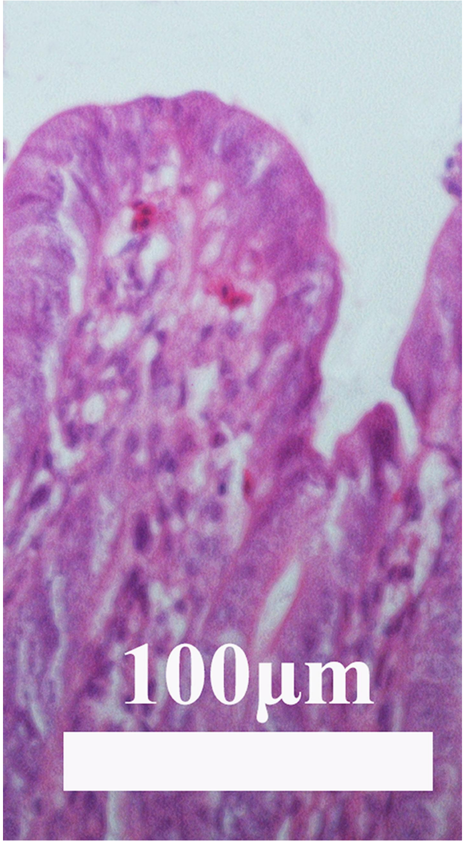

Fig. 4 Representative images of cecal histopathology from *E. tenella*-infected chicken at 72 hpi
